# Role of LrrkA in the Control of Phagocytosis and Cell Motility in *Dictyostelium discoideum*

**DOI:** 10.3389/fcell.2021.629200

**Published:** 2021-03-08

**Authors:** Romain Bodinier, Ayman Sabra, Jade Leiba, Anna Marchetti, Otmane Lamrabet, Imen Ayadi, Vedrana Filić, Takefumi Kawata, Igor Weber, Pierre Cosson

**Affiliations:** ^1^Department of Cell Physiology and Metabolism, Faculty of Medicine, University of Geneva, Geneva, Switzerland; ^2^Division of Molecular Biology, Ruder Boskovic Institute, Zagreb, Croatia; ^3^Department of Biology, Faculty of Science, Toho University, Tokyo, Japan

**Keywords:** LrrkA, folate, phagocytosis, motility, *Dictyostelium discoideum*

## Abstract

LrrkA is a *Dictyostelium discoideum* kinase with leucine-rich repeats. LrrkA stimulates Kil2 and intra-phagosomal killing of ingested bacteria in response to folate. In this study, we show that genetic inactivation of *lrrkA* also causes a previously unnoticed phenotype: *lrrkA* KO cells exhibit enhanced phagocytosis and cell motility compared to parental cells. This phenotype is cell autonomous, is reversible upon re-expression of LrrkA, and is not due to an abnormal response to inhibitory quorum-sensing factors secreted by *D. discoideum* in its medium. In addition, folate increases motility in parental *D. discoideum* cells, but not in *lrrkA* KO cells, suggesting that LrrkA plays a pivotal role in the cellular response to folate. On the contrary, *lrrkA* KO cells regulate gene transcription in response to folate in a manner indistinguishable from parental cells. Overall, based on analysis of mutant phenotypes, we identify gene products that participate in the control of intracellular killing, cell motility, and gene transcription in response to folate. These observations reveal a mechanism by which *D. discoideum* encountering bacterially-secreted folate can migrate, engulf, and kill bacteria more efficiently.

## Introduction

The initial contact between a cell and its substrate is a key element that determines the ability of the cell to adhere and spread on the substrate, and depending on its size to ingest it by phagocytosis (if it is small enough), or to move on its surface (Cougoule et al., 2004). Phagocytosis as well as cell adhesion, spreading, and motility involve massive changes in cell shape, which are carried out by a dynamic reorganization of the actin cytoskeleton. Actin-driven changes in cell shape can also lead to the enclosure of a large volume of extracellular medium, and its capture within an intracellular macropinosome, although in this situation the process of reshaping and ingestion is not driven by adhesion to a substrate (Buckley and King, 2017).

Phagocytosis is the process by which specialized mammalian cells (e.g., neutrophils and macrophages) and environmental amoebae ingest microorganisms, in particular bacteria (Bozzaro et al., 2008; Cosson and Soldati, 2008). Ingested bacteria are transferred from early phagosomes to acidic phago-lysosomes, where they are killed and digested. In mammals, one of the main functions of phagocytic cells is to destroy invading microorganisms and to protect the body against infections. Amoebae rather use phagocytosis to feed upon other microorganisms.

*Dictyostelium discoideum* has been an instrumental model to study the molecular mechanisms controlling the dynamics of the actin cytoskeleton, the phagocytic and endocytic pathway, and intracellular killing of bacteria (Cosson and Soldati, 2008; Mori et al., 2018; Stuelten et al., 2018). Due to the relative ease with which these haploid cells can be grown, observed, and genetically altered, they have proven instrumental to discover and analyze the role of multiple gene products in various facets of these cellular processes (Bretschneider et al., 2016; Buckley and King, 2017). *D. discoideum* has also proven useful to better understand human genetic diseases (McLaren et al., 2019) or to determine the mechanism of action of therapeutic drugs targeting a range of cellular mechanisms (Schaf et al., 2019).

Characterization of adhesion-defective *D. discoideum* mutant cells led to the discovery that SibA, a protein with integrin features, is a surface adhesion molecule necessary for efficient phagocytosis of certain substrates (Cornillon et al., 2006). Talin and myosin VII form a cytosolic complex (Tuxworth et al., 2005) that interacts with the cytosolic domain of SibA (Cornillon et al., 2006) and of several other cell surface adhesion molecules and is thus also necessary for efficient phagocytosis (Titus, 2005). Similarly, characterization of a mutant defective for intracellular killing of Klebsiella *pneumoniae* bacteria showed that Kil2, a putative magnesium pump in the phagosomal membrane, plays a key role in intraphagosomal killing of bacteria (Lelong et al., 2011). To the best of our current knowledge, molecular mechanisms involved in ingestion and killing of bacteria appear to be largely similar in *D. discoideum* and mammalian cells (Cosson and Soldati, 2008). *D. discoideum* has been used as a model system to isolate new bacteriolytic proteins (Dhakshinamoorthy et al., 2018) or anti-infective molecules of potential biomedical interest (Hanna et al., 2020).

Phagocytosis, phagosome maturation, and intracellular bacterial killing and digestion are complex processes involving multiple gene products (Pluddemann et al., 2011; Rosales and Uribe-Querol, 2017). Our understanding of the role of individual gene products in these processes is still largely incomplete. In addition, today, there is no unified model that would account for the diverse facets of phagocytosis and link it to other cellular functions that make use of similar mechanisms and gene products, notably macropinocytosis, cell motility, and intracellular signaling (Thomas et al., 2018; Pal et al., 2019; Vines and King, 2019).

In a recent study, we characterized a new *D. discoideum* mutant which kills ingested *K. pneumoniae* bacteria inefficiently (Bodinier et al., 2020). The *lrrkA* gene, inactivated in this mutant, encodes a kinase with leucine-rich repeats. Detailed analysis revealed that a putative signaling pathway implicating Far1 (the cell surface folate receptor), LrrkA, and Kil2 stimulates intracellular killing in response to extracellular folate.

In this study, we show that in addition to its role in killing, LrrkA also controls phagocytosis and cell motility. LrrkA is thus endowed with the ability to regulate coordinately cell motility, phagocytosis, and intracellular killing when *D. discoideum* cells are exposed to bacterially secreted folate.

## Results

### *lrrkA* KO Cells Phagocytose Particles More Efficiently Than WT Cells

**FIGURE 1:**
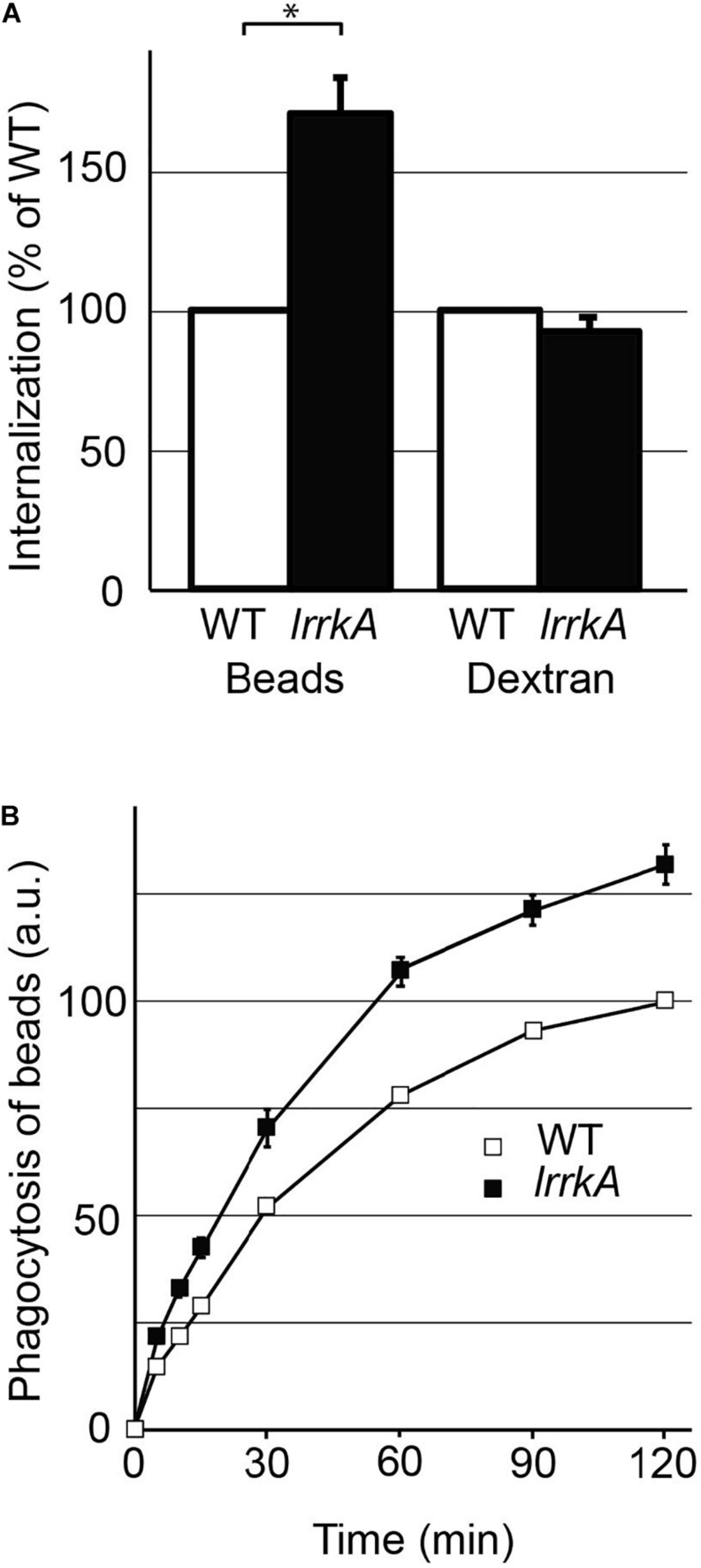
Genetic inactivation of *lrrkA* stimulates phagocytosis. **(A)** *D. discoideum* cells (WT or *lrrkA* KO) were incubated for 20 min in HL5 medium containing either fluorescent polystyrene beads, or a fluorescent dextran. Internalization of beads or of dextran was measured by flow cytometry in seven independent experiments and averaged. Internalization by *lrrkA* KO cells was expressed as the percentage of internalization by WT cells in the same experiment (mean ± SEM, ^∗^*p* < 0.01, Mann–Whitney test, *N* = 7 independent experiments). **(B)** *D. discoideum* cells (WT or *lrrkA* KO) were incubated for the indicated time in HL5 medium containing fluorescent polystyrene beads. The average and SEM at each time are indicated. *LrrkA* KO cells phagocytosed beads significantly more than WT cells at all times (*p* < 0.01, *N* = 9, Mann–Whitney test).

*LrrkA* KO cells were initially shown to kill inefficiently ingested *K. pneumoniae* bacteria (Bodinier et al., 2020). Beyond this defect, *lrrkA* KO cells did not exhibit any major alteration of the organization of the endocytic pathway: the overall structure of endocytic compartments appeared unchanged, as well as the acidic pH of lysosomes and phagolysosomes (Bodinier et al., 2020). Staining of the actin cytoskeleton also failed to reveal any gross anomaly of the actin cytoskeleton in *lrrkA* KO cells (Supplementary Figure S1). These observations left open, however, the possibility that kinetic parameters such as rates of endocytosis or of phagocytosis were modified in *lrrkA* KO cells. In order to measure the ability of *lrrkA* KO cells to perform phagocytosis, wild-type (WT) and *lrrkA* KO cells were incubated in the presence of fluorescent beads for 20 min in HL5 medium, and the number of internalized beads was then determined by flow cytometry (Figure 1A). *LrrkA* KO cells phagocytosed beads more efficiently than WT cells (171 ± 12% of WT). On the contrary, *lrrkA* KO cells did not ingest a fluid phase marker (fluorescent dextran) more efficiently than WT cells (93 ± 6% of WT). We also assessed phagocytosis of beads over a period of 2 h, and observed that *lrrkA* KO cells ingested beads more efficiently than WT cells at all times, even after 5 min of internalization (Figure 1B). The fact that phagocytosis was increased upon genetic inactivation of *lrrkA* while macropinocytosis was not, suggested that an increase in cell size was not responsible for the increased phagocytosis. We verified this by measuring directly cell size using two different techniques: image-based analysis (Tali cytometer) and electric current exclusion (CASY analyzer). Both techniques revealed that the size of *lrrkA* KO cells was essentially identical to that of WT cells: 100 ± 0.2% (average ± SEM; *N* = 3 independent experiments) for image analysis and 97.6 ± 0.2% (*N* = 3) for electric current exclusion.

### The Increased Phagocytosis of *lrrkA* KO Cells Is Cell Autonomous

Phagocytosis is a highly regulated process in *D. discoideum*. In particular, previous studies have shown that accumulation of (unidentified) quorum-sensing factors in the cell culture medium can inhibit significantly cell adhesion, phagocytosis, and cell motility in *D. discoideum* (Cornillon et al., 2008; Gole et al., 2011). A modification in the secretion of quorum-sensing factors could thus in principle result in a modification of phagocytosis rates.

We first studied whether the medium in which *lrrkA* KO cells grew contained less phagocytosis-inhibiting quorum-sensing factors than the medium in which WT cells grew. For this, we grew *lrrkA* KO or WT cells to the same density and collected the cell supernatants. WT cells were then exposed for 4 h to increasing concentrations of these two supernatants, and their rate of phagocytosis was measured. The rates of phagocytosis were identical for cells exposed to supernatants from *lrrkA* KO or from WT cells (Supplementary Figure S2), indicating that both supernatants contained the same amounts of phagocytosis-inhibiting quorum-sensing factors.

**FIGURE 2:**
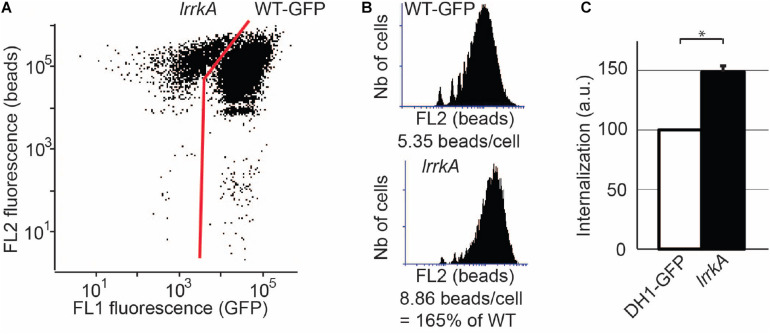
The phenotype of *lrrkA* KO cells is cell-autonomous. WT cells expressing GFP were mixed with *lrrkA* KO cells and co-cultured for 6 days. The mixed population of cells was then incubated with rhodamine-labeled polystyrene beads for 20 min, washed, and analyzed by flow cytometry. **(A)** Based on the GFP fluorescence, WT cells were easily distinguished from *lrrkA* KO cells. **(B)** Phagocytosis of beads was determined for WT and *lrrkA* KO cells. Even when mixed with WT cells, *lrrkA* KO cells exhibited more phagocytic activity than WT cells. In the experiment shown, *lrrkA* KO cells ingested 1.65 times more beads than WT cells. **(C)** The single experiment shown in **A** and **B** was repeated and quantified (^∗^*p* < 0.01, Mann–Whitney test; *N* = 6 independent experiments).

We further tested whether the phenotype of *lrrkA* KO cells is cell-autonomous. For this, we mixed and co-cultured WT cells expressing GFP and *lrrkA* KO cells for 6 days. We then incubated the mixed population with rhodamine-labeled polystyrene beads, and analyzed uptake of beads by flow cytometry. Since GFP-expressing WT cells can readily be distinguished from *lrrkA* KO cells based on the fluorescence of GFP (Figure 2A), the phagocytosis of beads was analyzed for the two populations of cells (Figure 2B). The single experiment shown in Figures 2A,B was repeated and quantified: *lrrkA* KO cells mixed with GFP-expressing WT cells exhibited a higher level of phagocytosis (150 ± 4% of WT) (Figure 2C). This result indicates that the phenotype of *lrrkA* KO cells is cell-autonomous and does not reflect differences in the conditions (medium, cell density, contact with other cells…) in which the cells are grown.

**FIGURE 3:**
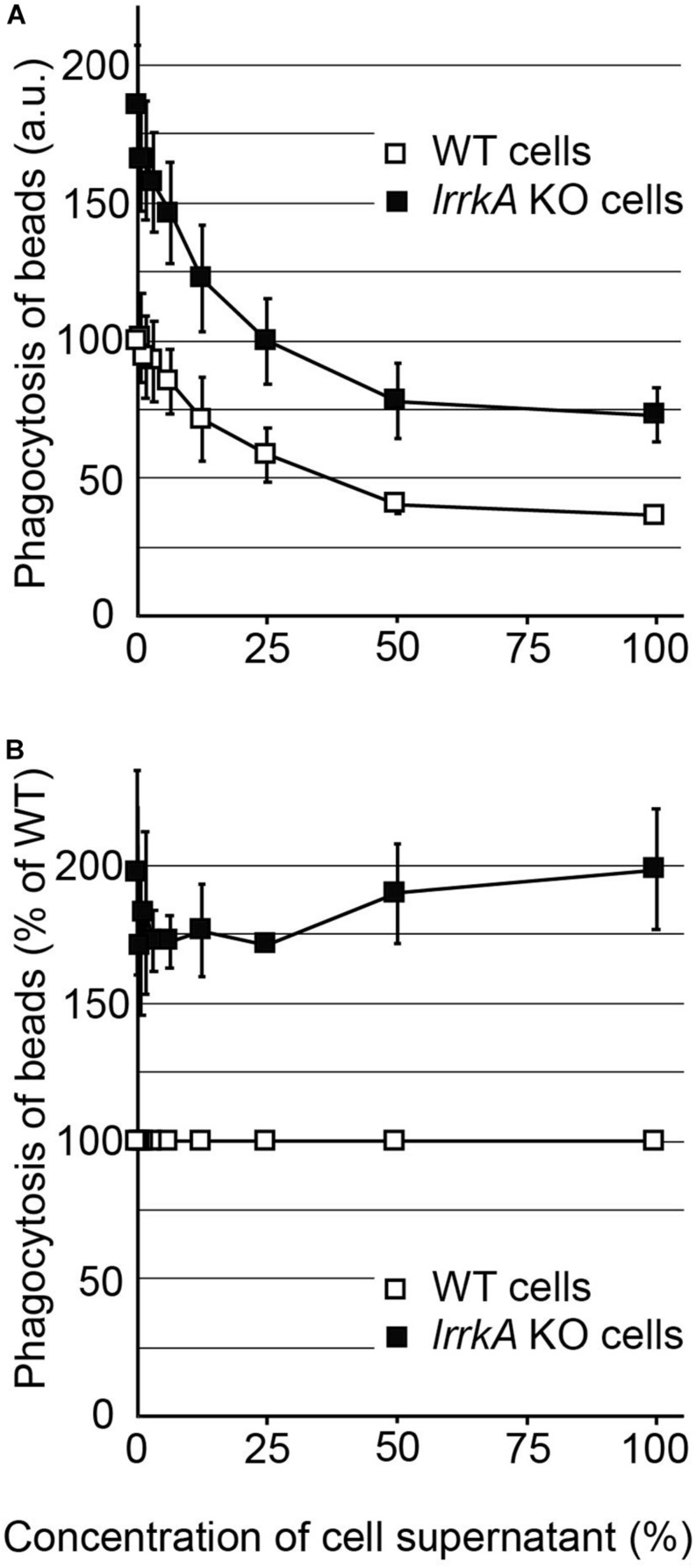
Response of *lrrkA* KO cells to secreted quorum-sensing factors. **(A)** WT or *lrrkA* KO cells were incubated for 4 h with a cellular supernatant of WT cells diluted in fresh medium as indicated. The ability of the cells to ingest polystyrene beads was then measured as described in the legend to Figure 1. At all concentrations of quorum-sensing factors, *lrrkA* KO cells phagocytosed beads more efficiently than WT cells (average ± SEM, *N* = 3 independent experiments). **(B)** For each condition, phagocytosis by *lrrkA* KO cells was represented as a percentage of WT phagocytosis in the same condition. In all conditions, *lrrkA* KO cells phagocytosed 1.5–2 times more beads than WT cells.

A modification in the cellular response to quorum-sensing factors could also in principle result in a cell-autonomous modification of phagocytosis rates. To test this possibility, we exposed *lrrkA* KO and WT cells for 4 h to an increasing concentration of quorum-sensing factors secreted by WT cells; then we measured their ability to perform phagocytosis. At all concentrations of quorum-sensing factors tested, including in the absence of quorum-sensing factors, *lrrkA* KO cells phagocytosed more efficiently than WT cells (Figure 3A). Moreover, when the rates of phagocytosis were compared between *lrrkA* KO and WT cells incubated in the same conditions, the phenotype was quantitatively virtually identical in all conditions, with *lrrkA* KO cells ingesting approximately 1.7 times more beads than WT cells (Figure 3B). These observations indicate that *lrrkA* KO cells respond normally to secreted quorum-sensing factors.

**FIGURE 4:**
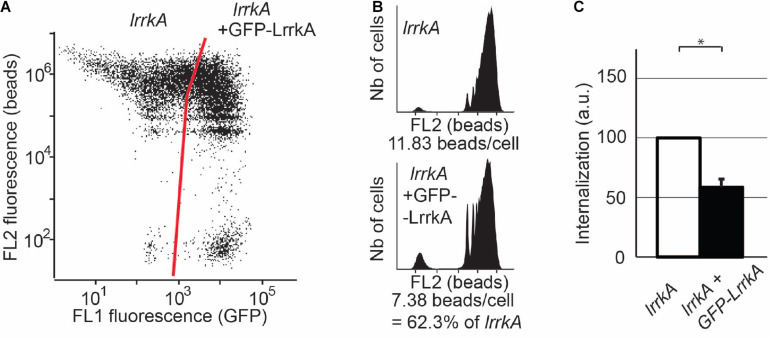
Reexpression of LrrkA decreases phagocytosis in *lrrkA* KO cells. **(A)** Expression of GFP-LrrkA in *lrrkA* KO cells was assessed by flow cytometry. After 5 days of culture with no G418 selection, approximately 75% of transfected cells exhibited detectable GFP-LrrkA, while 25% did not produce the protein. **(B)** Phagocytosis was assessed by flow cytometry in *lrrkA* KO cells expressing GFP-LrrkA and in non-expressing cells. **(C)** Phagocytosis in *lrrkA* KO cells expressing GFP-LrrkA was measured in five independent experiments and expressed as a percentage of phagocytosis in cells that did not express GFP-LrrkA (mean ± SEM, ^∗^*p* < 0.05, Mann–Whitney test, *N* = 5 independent experiments).

Finally, in order to verify that the increased phagocytosis observed in *lrrkA* KO cells resulted from the genetic inactivation of *lrrkA*, we introduced in these cells a plasmid producing GFP-tagged LrrkA. Approximately 75% of the transfected cells expressed detectable levels of GFP-LrrkA (Figure 4A). The fusion protein was localized mostly in the nucleus and cytosol, with no clear accumulation at the level of cellular membranes (Supplementary Figure S3). Phagocytosis in cells expressing GFP-LrrkA was compared to phagocytosis in non-expressing cells (Figure 4B). We observed that expression of GFP-LrrkA in *lrrkA* KO cells decreased phagocytosis (Figure 4C), which demonstrates that the increased phagocytosis in *lrrkA* KO cells was indeed caused by the loss of LrrkA in these cells.

### LrrkA Controls Interaction of *D. discoideum* With Its Substrate

The phagocytosis experiments described above take place in HL5 medium. In these conditions, SibA is the phagocytic receptor responsible for phagocytosis of beads, while it is dispensable in phosphate buffer where other (unidentified) receptors are engaged (Benghezal et al., 2003). We compared by western blot the amount of SibA found in *lrrkA* KO and WT cells, and did not detect any significant difference (Supplementary Figure S4).

**FIGURE 5:**
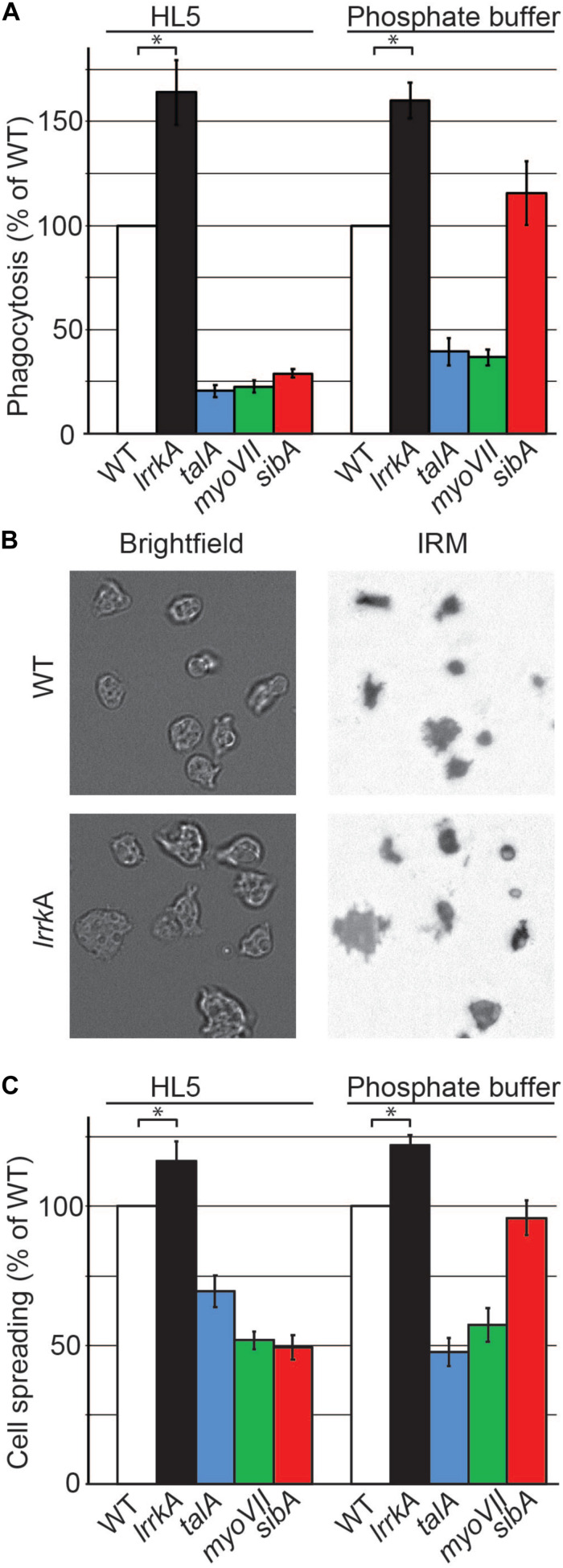
Genetic inactivation of *lrrkA* increases interaction with substrates in HL5 and in phosphate buffer. **(A)** Phagocytosis of polystyrene beads was measured for the indicated mutant cells as described in the legend to Figure 1. *LrrkA* KO cells phagocytosed more efficiently than WT cells both in HL5 and in phosphate buffer (PB) (average ± SEM; ^∗^*p* < 0.01, Mann–Whitney test; *N* = 19 independent experiments). **(B)** Brightfield and interference reflection pictures of WT and *lrrkA* KO cells (here in HL5) allow to visualize the contact area between cells and their substratum. **(C)** Spreading of WT and mutant cells on their substrate in HL5 and in PB reflects their ability to phagocytose particles. The contact surface between WT cells and the glass substrate was 62 and 87 μm^2^ in HL5 and SB, respectively. Spreading of all mutants analyzed was significantly different from WT, except for *sibA* KO cells in phosphate buffer (average ± SEM; ^∗^*p* < 0.01, Student’s *t*-test, number of cells analyzed in HL5/SB: WT 197/162; *lrrkA* 199/85; *talA* 173/125; *myoVII* 242/79; *sibA* 202/121; *N* = 2 independent experiments).

We then measured phagocytosis in phosphate buffer for WT and *lrrkA* KO cells. *LrrkA* KO cells phagocytosed more efficiently than WT cells both in HL5 medium (164 ± 16% of WT) and in phosphate buffer (PB) (160 ± 8% of WT) (Figure 5A). As expected, genetic inactivation of Talin A (*talA*) and Myosin VII (*myoVII*) also inhibited phagocytosis in both HL5 medium and PB, while genetic inactivation of SibA inhibited phagocytosis only in HL5 (Figure 5A). Thus, phagocytosis of beads is increased in *lrrkA* KO cells in PB, i.e., in conditions where SibA does not play a role in phagocytosis. In summary, the increased phagocytosis of *lrrkA* KO cells is not caused by an increase in the amount or activity of SibA.

In many mutants, changes in the phagocytic process result from alterations in the interaction between the phagocytosed particle and the phagocytic cell, for example, an alteration in the ability of cells to adhere to a particle or to spread on its surface. However, the phagocytic event is transient and difficult to visualize, and it is much easier to characterize the interaction of *D. discoideum* with a flat surface. In order to measure the ability of *lrrkA* KO cells to interact with a substrate, we visualized and measured their zone of tight contact with a glass surface by interference reflection microscopy (Figure 5B). Quantitative analysis revealed that *lrrkA* KO cells spread more efficiently than WT cells on the substrate, both in HL5 and in phosphate buffer (Figure 5C). As expected, *talA* and *myoVII* KO cells exhibited defective spreading in both HL5 and phosphate buffer, while *sibA* KO cells spread less efficiently than WT cells in HL5, but normally in phosphate buffer (Figure 5C).

Together these results suggest that LrrkA negatively regulates the cytosolic machinery engaged in cellular adhesion and phagocytosis.

### LrrkA Plays a Role in the Control of Cell Motility

**FIGURE 6:**
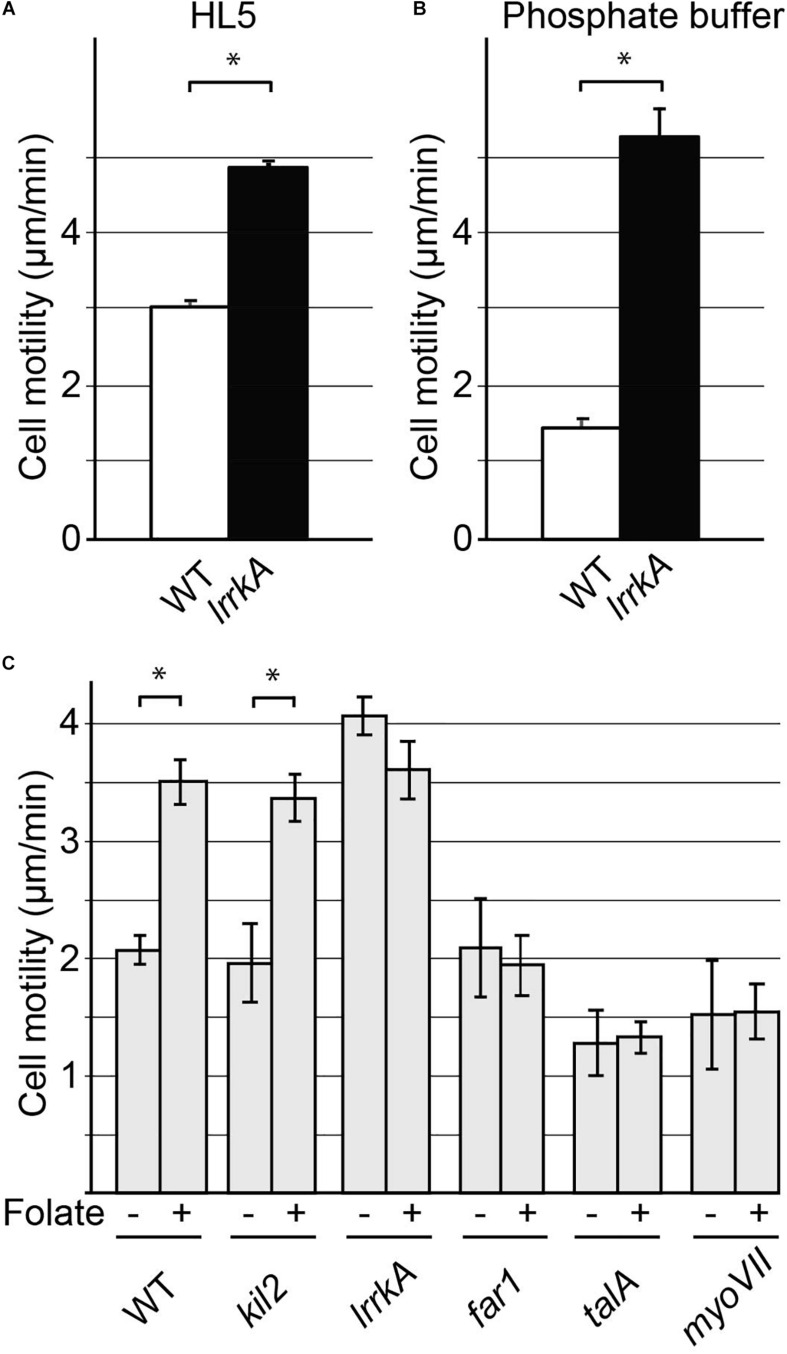
Cell motility is increased by genetic inactivation of *lrrkA*. Random motility of WT or *lrrkA* KO cells on a substrate was measured in HL5 **(A)** and phosphate buffer **(B)**. Motility was significantly higher in *lrrkA* KO cells than in WT cells (average ± SEM; ^∗^*p* < 0.01, Student’s *t*-test, number of cells analyzed in HL5/SB: WT 110/195; *lrrkA* 363/225; *N* = 3 independent experiments). Random motility of WT or mutant cells was also measured in phosphate buffer in the presence or absence of folate **(C)**. Motility increased significantly in WT cells or in *kil2* KO cells upon exposure to folate. In *lrrkA* KO cells, cell motility was high even in the absence of folate, and was not further stimulated upon folate addition. In *far1*, *talA*, and *myoVII* KO cells, folate failed to stimulate cell motility (average ± SEM; ^∗^*p* < 0.01, Student’s *t*-test, number of cells analyzed in SB/SB + folate: WT 225/90; *lrrkA* 315/150; *kil2*, far1, *talA, myoVII* 45/45; *N* = 3 independent experiments). Distinct experiments are reported in **(B)** and **(C)**.

To characterize in a more kinetic manner the interaction between cells and their substrate, we measured the ability of cells to move on their substrate. We first determined the random motility of WT and *lrrkA* KO cells on a glass substrate in HL5 by taking pictures every 10 seconds. This revealed a significant increase in motility of *lrrkA* KO cells compared to WT cells (Figure 6A). We also observed a major increase in motility of *lrrkA* KO cells compared to WT cells when analyzed in phosphate buffer (Figure 6B).

Our recent results suggested that LrrkA participates in a putative signaling pathway linking the Far1 folate receptor to Kil2 and allowing folate to stimulate intracellular killing (Bodinier et al., 2020). We thus tested similarly the ability of various mutant cells to modify their motility in response to folate. For this, we measured random cell motility in phosphate buffer supplemented or not with 1 mM folate. As previously described (Lima et al., 2014), folate stimulated motility of WT and of *kil2* KO cells (Figure 6C). Unstimulated *lrrkA* KO cells were more mobile than WT cells, but their motility did not increase further upon addition of folate (Figure 6C). Unstimulated *far1* KO cells, devoid of folate receptor, exhibited normal motility but did not respond to the addition of folate (Figure 6C). In *talA* or *myoVII* KO cells, the unstimulated motility was reduced, and the cells failed to respond to folate (Figure 6C). As discussed below, the fact that motility is higher in *lrrkA* KO cells and that it is unchanged upon addition of folate indicates that LrrkA plays a role in the control of cell motility in *D. discoideum* cells.

### LrrkA Is Dispensable for the Transcriptional Response to Folate

**FIGURE 7:**
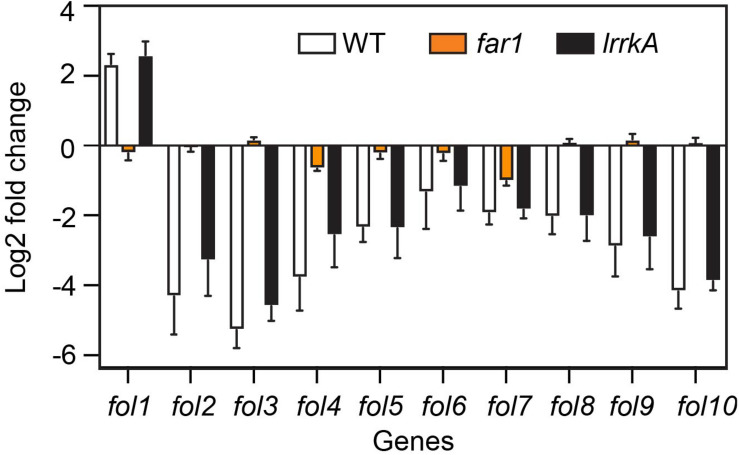
Folate control of gene transcription is independent of LrrkA. We measured by qPCR the levels of various RNAs in cells exposed or not to folate. The average and SEM of three independent experiments are shown. As previously observed, the transcription of genes *fol1*-*fol10* is altered when cells are exposed to folate (increased for *fol1*, decreased for *fol2-10*). This regulation is essentially lost in *far1* KO cells. On the contrary, in *lrrkA* KO cells, transcriptional response to folate is indistinguishable from that observed in WT cells.

We recently studied by RNA sequencing the transcription profiles of *D. discoideum* cells exposed to various stimuli, in particular folate (Lamrabet et al., 2020). This allowed us to define a set of ten genes whose expression varies specifically upon exposure to folate (Supplementary Figure S5A). We first validated these results by preparing RNA from WT *D. discoideum* cells exposed to folate or not. This experiment confirmed that the expression of the selected set of genes is regulated by folate (Supplementary Figure S5B). We then measured the expression of these genes in WT, *far1*, or *lrrkA* KO cells (Figure 7). In *far1* KO cells, gene expression was not modified by exposure to folate, confirming that folate sensing relies critically on the Far1 receptor. On the contrary, *lrrkA* KO cells responded to folate in a manner indistinguishable from WT cells (Figure 7), indicating that LrrkA does not play a critical role in gene regulation upon folate exposure.

## Discussion

**FIGURE 8:**
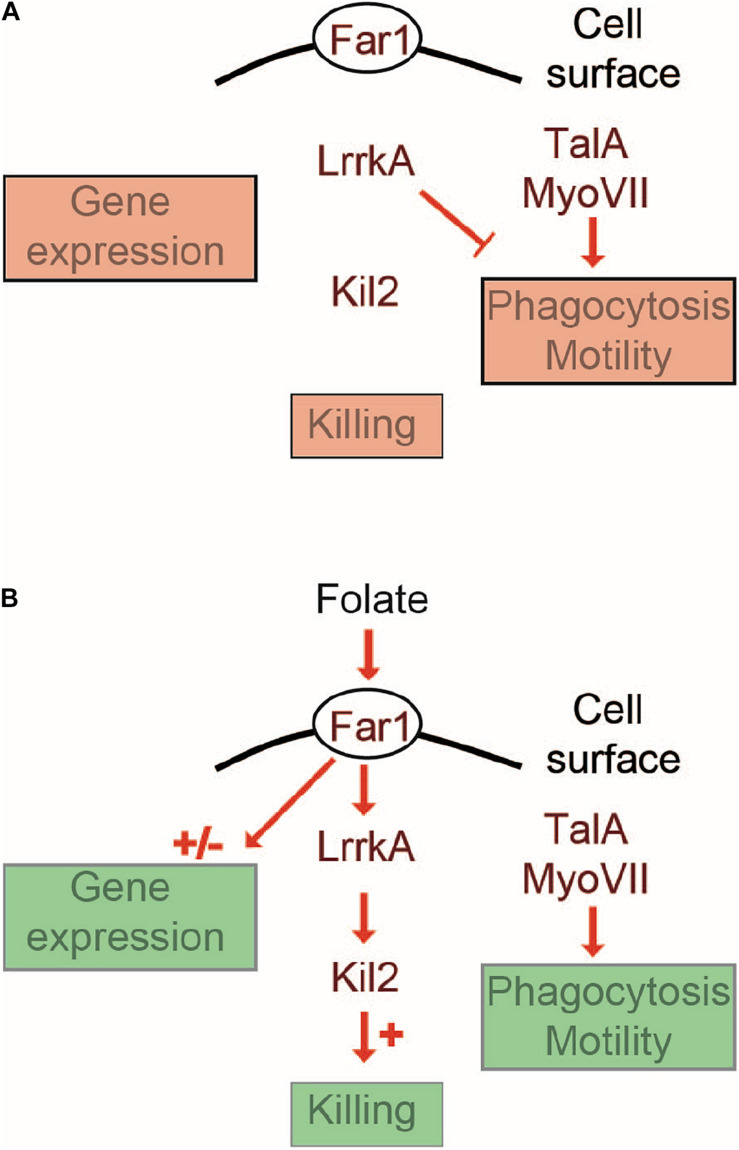
Cellular response to folate: a working model. This schematic overview proposes a minimalist interpretation of the results presented in this manuscript and in previous publications. Far1, the cell surface receptor for folate is essential for all cellular responses to folate. A Far1-LrrkA-Kil2 pathway stimulates intracellular killing in response to folate. LrrkA inhibits cell motility in the absence of folate **(A)**, and the inhibition is relieved upon stimulation by folate **(B)**. The transcriptional response to folate operates in an LrrkA-independent manner.

In this study, we describe a new role for LrrkA as a negative regulator of cell motility and phagocytosis. Based on these observations and on the previously published role of LrrkA in activation of intracellular killing (Bodinier et al., 2020), we propose a model where LrrkA functions in two modes, as schematized in Figure 8. In the absence of folate (Figure 8A), LrrkA does not stimulate killing. It inhibits motility and phagocytosis, as evidenced by the fact that genetic inactivation of *lrrkA* leads to an increase in phagocytosis and motility in these conditions. In the presence of a high concentration of folate (Figure 8B), which in the environment is secreted by bacteria, Far1 activates LrrkA which in turn stimulates intracellular killing via activation of Kil2, as previously reported (Bodinier et al., 2020). In the same conditions, the inhibitory effect of LrrkA on motility is lost, as evidenced by the fact that *lrrkA* KO cells are as motile as parental cells. According to this model, the ability of folate to increase cell motility would at least partly be due to the fact that it relieves the inhibition of cell motility by LrrkA. This rapid LrrkA-dependent adaptation of cellular physiology to changes in the environment should be distinguished from slower adaption involving changes in gene transcription which, according to our observations, does not rely on the function of LrrkA. Our study does not provide biochemical evidence demonstrating direct physical interaction of LrrkA with other proteins, or identifying the proteins that are phosphorylated by LrrkA. The proposed model is the simplest model compatible with the observed phenotype of mutant cells, but many alternative models could be proposed. Whatever the molecular organization of these signaling pathways, our results suggest that LrrkA plays an important role in allowing *D. discoideum* cells to increase coordinately motility, phagocytosis, and intracellular killing when they sense the presence of bacterial folate, thus favoring both an efficient capture and intracellular killing of bacteria. We are aware that intracellular signaling pathways are often more branched and complex than the simple scheme proposed in this study. More detailed studies will certainly be necessary to determine the exact role of LrrkA in the hierarchy of intracellular signaling pathways controlling cell motility, phagocytosis, and intracellular killing.

*Dictyostelium discoideum* has been used as a simple model system to analyze the mode of action of bioactive compounds like lithium and valproic acid (two mood-stabilizing drugs), or to discover new compounds with similar modes of action (Schaf et al., 2019). Similarly, *D. discoideum* has proven valuable to study the molecular basis for lysosomal diseases like the neuronal ceroid lipofuscinoses (McLaren et al., 2019). In these two instances, a detailed understanding of the underlying molecular mechanisms was an essential prerequisite to allow extrapolation of the results from *D. discoideum* to human. Human LRRK2 kinase has been linked to the function of the endocytic pathway, but its function is still incompletely understood, as well as its presumptive role in the etiology of sporadic Parkinson’s disease (Bodinier et al., 2020). It is difficult at this stage to establish a parallel between the molecular function(s) of Lrr kinases in human and in *D. discoideum*, since the family of Lrr kinases is much less diverse in human than in *D. discoideum* (Bodinier et al., 2020). However, our results suggest that LrrkA, like LRRK2, is linked to the regulation of the endocytic pathway in *D. discoideum*. Hopefully, these observations will contribute to the development of *D. discoideum* as a tractable model system for biomedical research.

## Materials and Methods

### Strains and Cell Culture

*Dictyostelium discoideum* cells were grown at 21°C in HL5 medium (Froquet et al., 2009). *D. discoideum* cells were all derived from the DH1-10 parental strain (Cornillon et al., 2000), referred to in this study as WT. *lrrk*A KO, *far*1 KO, *kil*2 KO, *sib*A KO, *tal*A KO, and *myo*VII KO cells were described previously (Gebbie et al., 2004; Tsujioka et al., 2008; Lelong et al., 2011; Froquet et al., 2012; Pan et al., 2016; Bodinier et al., 2020). In order to re-express GFP-LrrkA in *lrrkA* KO cells, we cloned the coding sequence of LrrkA in the pDM351 expression vector (Veltman et al., 2009), which drives the production of LrrkA fused at its N-terminal end to the GFP protein. The transfected cells were selected in the presence of G418 (12.5 μg/mL). They were transferred to HL5 without G418 5 days before testing their ability to perform phagocytosis.

### Phagocytosis and Macropinocytosis

To measure efficiency of phagocytosis, 3 × 10^5^ *D. discoideum* cells were washed once, resuspended in either 1 mL of HL5 or 1 mL of phosphate buffer (PB: 2 mM Na_2_HPO_4_, 14.7 mM KH_2_PO_4_, pH 6.5) containing 1 μl of FITC polystyrene beads (Fluoresbrite plain YG 1 micron, Polysciences), and incubated for 30 min in a shaken suspension. To assess macropinocytosis, cells were incubated in either HL5 or PB containing 10 μg/mL Alexa-647 Dextran (Life Technologies) for 30 min. Then, cells were washed in ice cold HL5 supplemented with 0.1% NaN_3_ and internalized fluorescence was measured by flow cytometry. Ingested fluorescence was determined for each strain and normalized to internalization in WT cells.

For phagocytosis kinetic measurements, cells were incubated in 1 mL of HL5 containing 1 μL FITC polystyrene beads, and a 100 μL aliquot of the suspension was taken at each indicated time point. Cells were then washed, and phagocytosis was analyzed as described above.

When mixed populations containing GFP-expressing cells (FL1 channel) were used, they were allowed to ingest red fluorescent beads (Fluoresbrite Polychromatic Red, Polysciences, FL2 channel). The fluorescence values were corrected to compensate for leakage of fluorescence between the FL1 and FL2 channels. To ascertain that the cells were adequately identified as GFP-positive or -negative, control populations were analyzed. When *lrrkA* KO cells were mixed with WT-GFP cells (Figure 2), *lrrkA* KO and WT-GFP cells were analyzed separately in parallel, and each cell type was present almost exclusively in the assigned window (99.9 and 99.5%, respectively). Similarly, when *lrrkA* KO cells were transfected with a GFP-LrrkA expressing plasmid, untransfected cells were analyzed in parallel and were almost exclusively (99.8%) found in the GFP-negative window. In the transfected population, GFP-negative cells represented 24% of the total in a control sample (no beads phagocytosed), and 23.6% in the sample of cells having ingested beads, indicating that no significant number of cells were assigned to the wrong population. In all measures of phagocytosis, median values were used, ensuring that a small percentage of cells improperly identified as GFP-negative or GFP-positive would not significantly affect the results.

To produce conditioned cellular supernatant, *D. discoideum* cells were grown to a density of 4 × 10^6^ cells in a shaken suspension in HL5 medium. The cell supernatant was recovered by centrifuging cells at 1,500 *g* for 10 min. To assess the effect of conditioned medium on phagocytosis, cells were incubated for 4 h in fresh or conditioned medium in six-well plates (450,000 cells per well in 1.5 mL), then resuspended, and allowed to phagocytose beads for 5 min in fresh HL5 medium.

### Cell Volume Measurement

Cell size was determined based on electric current exclusion (CASY technology), using a CASY 1 cell counter as previously described (Dias et al., 2016). The Tali image-based cytometer (ThermoFischer Scientific) was also used to automatically measure the size of cells based on pictures of cell suspension.

### Cellular Motility

To assess the motility of *D. discoideum* cells, 10^5^ cells were washed once and resuspended in 1 mL of PB/Sorbitol (PB + 100 mM Sorbitol). 100 μL of the cell suspension was deposited in 96-well plates (Greiner Bio one; Ref 655090), and the cells allowed to settle for 30 min. Then 100 μL of PB/Sorbitol supplemented or not with 1 mM folate was added to each well. Cells were imaged every 15 s for 30 min with a Nikon Eclipse T*i*2 equipped with a DS-Qi2 camera. The resulting movies were analyzed with the software MetaMorph (Molecular Devices) using the “Track points” function.

### Cell Spreading

To measure the spreading of *D. discoideum* cells on a surface, 5 × 10^5^ cells were washed once and resuspended in either 1 mL HL5 or 1 mL PB. A 50 μL drop was deposited on a glass-bottom fluorodish (WPI, Inc.; ref: FD35-100), and the cells allowed to attach for 30 min. A Zeiss Axio Observer Z1 equipped with a Neofluar 63x/1.25 Oil Ph3 Antiflex objective for RICM measurement was used for imaging. Quantification of the cell speading surface was done using FiJi (v1.52j).

To visualize the actin cytoskeleton, 10^6^ *D. discoideum* cells were let to adhere to a glass coverslip for 30 min in HL5 medium. Cells were then fixed with 4% paraformaldehyde for 30 min, washed, permeabilized with methanol at −20°C for 2 min, and labeled with 1 μg/mL tetramethylrhodamine B isothiocyanate (TRITC)-coupled phalloidin in PBS containing 0.2% bovine serum albumin (PBS-BSA) for 1 h. The coverslips were washed twice in PBS-BSA, once in PBS, then mounted, and observed by laser scanning confocal microscopy (Zeiss LSM 800).

### Western Blot

To determine the levels of cellular proteins, 10^6^ cells were resuspended in 10 μL of sample buffer (0.103 g/mL sucrose, 50 mM Tris, pH 6.8, 10 mM EDTA, 0.5 mg/mL bromophenol blue, 2% SDS), and proteins were separated by electrophoresis on an SDS-polyacrylamide gel. Proteins were then transferred to a nitrocellulose membrane before immunodetection with anti-SibA (Cornillon et al., 2006), anti-TalinA (Benghezal et al., 2006), or anti-protein disulfide isomerase (PDI) (Lelong et al., 2011) primary antibodies. Horseradish-peroxidase-coupled anti-mouse (for anti-TalinA and anti-PDI) and anti-rabbit (for anti-SibA) antibodies were used as secondary antibodies. The signal was revealed by enhanced chemiluminescence (ECL) (Amersham Biosciences) using a PXi-4 gel imaging systems (Syngene).

### Reverse-Transcription Quantitative PCR (RT-PCR)

Reverse-transcription (RT)-PCR experiments were performed essentially as previously described (Lamrabet et al., 2020). *D. discoideum* cells (4 × 10^6^ cells/mL) were incubated for 4 h in HL5 supplemented or not with 1 mM folate. The total RNAs from *D. discoideum* or mutant cells exposed or not to 1 mM folate were purified with a Qiagen RNeasy kit following manufacturer’s instructions. cDNA was synthesized from 1 μg of total RNA using random hexamers and Superscript II reverse transcriptase (Invitrogen).

For each gene analyzed, oligonucleotide sequences were aligned against the *D. discoideum* coding sequence database by BLAST to ensure that they were specific for the gene tested. PCR reactions (10 μL) contained SYBR Green Master Mix (Applied Biosystems), diluted cDNA (150 ng), and 500 nM of forward and reverse oligonucleotides, and were analyzed in a StepOnePlus cycler (Invitrogen) with the following parameters: 95°C for 1 min, 40 cycles of 95°C/10 s, and 60°C/1 min. Twofold changes were calculated as Δ(ΔCT), with the *rnlA* and *gpdA* genes used as a standard for normalization. Data were collected from three biological replicates.

## Data Availability Statement

The raw data supporting the conclusions of this article will be made available by the authors, without undue reservation.

## Author Contributions

RB, AS, JL, AM, OL, IA, and VF designed and performed the experiments and interpreted the data. RB and PC wrote the manuscript. TK, IW, and PC supervised the project. All authors reviewed the manuscript.

## Conflict of Interest

The authors declare that the research was conducted in the absence of any commercial or financial relationships that could be construed as a potential conflict of interest.

## Supplementary Material

Supplementary Figure S1 Morphology of the actin cytoskeleton in WT and *lrrkA* KO cells. WT and *lrrkA* KO were allowed to adhere to a glass coverslip, then fixed, permeabilized, and stained with fluorescent phalloidin to reveal the structure of the actin cytoskeleton. Three pictures are shown for WT and for *lrrkA* KO cells. The morphology of the actin cytoskeleton appeared highly similar in WT and *lrrkA* KO cells. Bar: 10 μm.

Supplementary Figure S2 Secretion of quorum-sensing factors by *lrrkA* KO cells. WT cells were incubated for 4 h with cell supernatants from WT or *lrrkA* KO cells diluted as indicated with fresh HL5. The cells were then incubated with polystyrene beads and phagocytosis measured as described in the legend to Figure 1. Phagocytosis was expressed as the percentage of internalization by WT cells in fresh medium in the same experiment (average ± SEM, *N* = 3 independent experiments). Cells incubated in fresh medium (0% cell supernatant) phagocytosed beads approximately four times more efficiently than cells incubated in pure cell supernatants (100% cell supernatant) containing concentrated quorum-sensing factors. Cell supernatants diluted with fresh medium were also assessed to evaluate more precisely the quorum-sensing activity. The supernatant of *lrrkA* KO cells exhibited exactly the same inhibitory effect on phagocytosis as the supernatant of WT cells.

Supplementary Figure S3 GFP-LrrkA is localized in the nucleus and the cytosol. *LrrkA* KO cells expressing GFP-LrrkA were fixed with 2% formaldehyde and visualized by confocal microscopy. GFP fluorescence was visible in the nucleus (arrowheads) and in the cytosol. Untransfected *lrrkA* KO cells were used in parallel as negative controls and did not show any GFP fluorescence. Bar: 5 μm.

Supplementary Figure S4 Cellular levels of SibA, PDI, and Talin A are similar in WT and *lrrkA* KO cells. **(A)** Cellular proteins were separated on an SDS-polyacrylamide gel, transferred to nitrocellulose, and the indicated proteins were detected with specific antibodies. **(B)** The intensity of the signal was determined in several independent experiments, and is indicated as a ratio of the signal in *lrrkA* KO and WT cells. The quantification of the experiment shown in **A** is indicated in red. The amount of SibA, Talin, and PDI (used as a control) was indistinguishable in *lrrkA* KO and WT cells.

Supplementary Figure S5 Regulation of gene expression by folate. *D. discoideum* WT cells grown in HL5 were exposed or not to 1 mM folate for 4 h. RNA-seq analysis allowed the identification of 10 genes for which transcription was significantly altered by exposure to folate. **(A)** For each gene considered (*fol1* to *fol10*), the gene identity and name are indicated, as well as the pair of primers used for RT-PCR and the size of the amplicon. **(B)** For the 10 genes analyzed, RT-PCR analysis confirmed the results obtained by RNA-seq.
