# Supplementary material for: Role of LrrkA in the Control of Phagocytosis and Cell Motility in Dictyostelium discoideum
Source: Front Cell Dev Biol. 2021 Mar 8;9:629200. doi: 10.3389/fcell.2021.629200 (PMC7982419; doi:10.3389/fcell.2021.629200)
Supplement: Supplementary Figure S1 — Morphology of the actin cytoskeleton in WT and lrrkA KO cells. WT and lrrkA KO were allowed to adhere to a glass coverslip, then fixed, permeabilized, and stained with fluorescent phalloidin to reveal the structure of the actin cytoskeleton. Three pictures are shown for WT and for lrrkA KO cells. The morphology of the actin cytoskeleton appeared highly similar in WT and lrrkA KO cells. Bar: 10 μm. [file Image_1.pdf]

SUPPLEMENTARY FIGURES

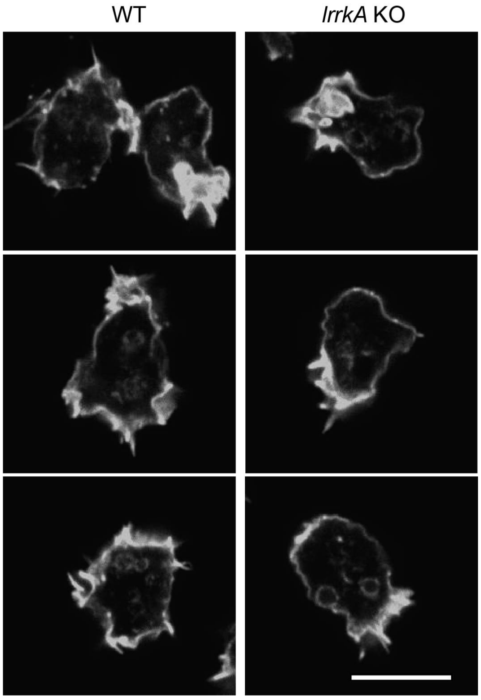

Figure S1. Morphology of the actin cytoskeleton in WT and *lrrkA* KO cells. WT and *lrrkA* KO were allowed to adhere to a glass coverslip, then fixed, permeabilized and stained with fluorescent phalloidin to reveal the structure of the actin cytoskeleton. Three pictures are shown for WT and for *lrrkA* KO cells. The morphology of the actin cytoskeleton appeared highly similar in WT and *lrrkA* KO cells. Bar: 10µm.

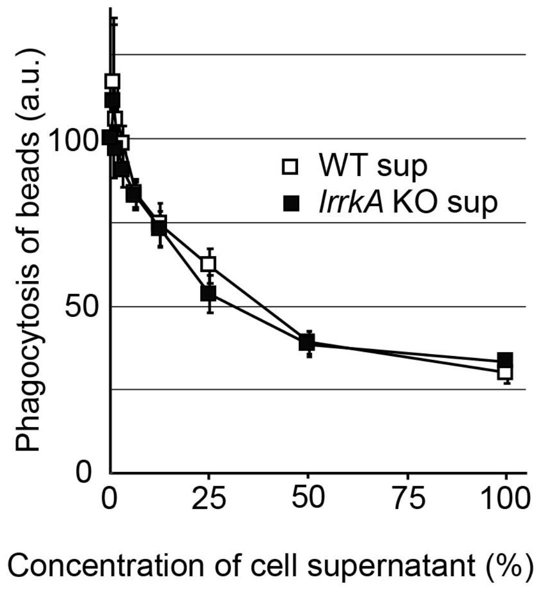

Figure S2. Secretion of quorum-sensing factors by *lrrkA* KO cells. WT cells were incubated for 4h with cell supernatants from WT or *lrrkA* KO cells diluted as indicated with fresh HL5. The cells were then incubated with polystyrene beads and phagocytosis measured as described in the legend to figure 1. Phagocytosis was expressed as the percentage of internalization by WT cells in fresh medium in the same experiment (average ± s.e.m., N=3 independent experiments). Cells incubated in fresh medium (0% cell supernatant) phagocytosed beads approximately four times more efficiently than cells incubated in pure cell supernatants (100% cell supernatant) containing concentrated quorum-sensing factors. Cell supernatants diluted with fresh medium were also assessed to evaluate more precisely the quorum-sensing activity. The supernatant of *lrrkA* KO cells exhibited exactly the same inhibitory effect on phagocytosis as the supernatant of WT cells.

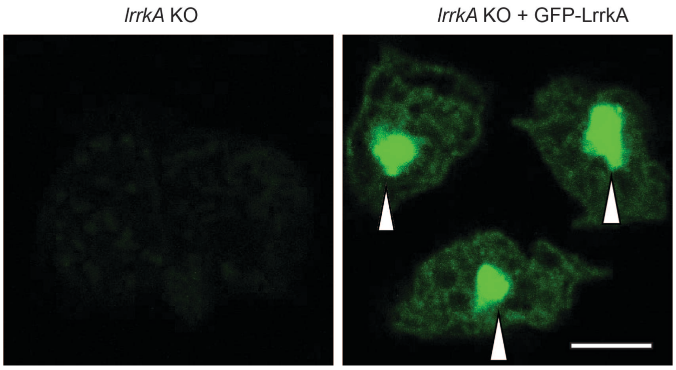

Figure S3. GFP-LrrkA is localized in the nucleus and the cytosol. *LrrkA* KO cells expressing GFP-LrrkA were fixed with 2% formaldehyde and visualized by confocal microscopy. GFP fluorescence was visible in the nucleus (arrowheads) and in the cytosol. Untransfected *lrrkA* KO cells were used in parallel as negative controls and did not show any GFP fluorescence. Bar: 5μm.

A

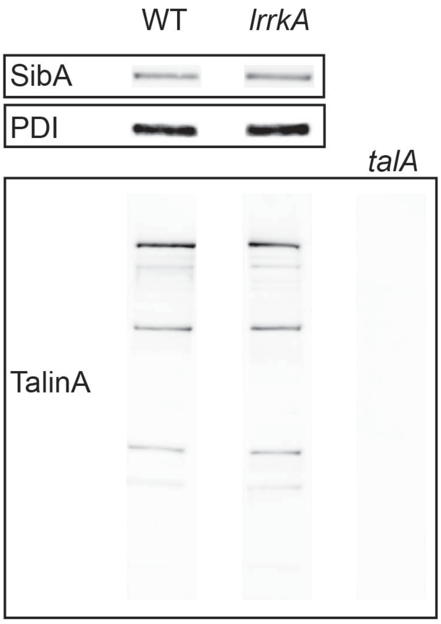

B

	***lrrkA*/ WT**
**SibA**	0.82
	1.60
	1.23
	1.07
**Talin**	0.88
	1.01
**PDI**	0.93
	1.03
	1.09

Figure S4. Cellular levels of SibA, PDI and Talin A are similar in WT and *lrrkA* KO cells. A. Cellular proteins were separated on an SDS-polyacrylamide gel, transferred to nitrocellulose, and the indicated proteins were detected with specific antibodies. B. The intensity of the signal was determined in several independent experiments, and is indicated as a ratio of the signal in *lrrkA* KO and WT cells. The quantification of the experiment shown in A are indicated in red. The amount of SibA, Talin and PDI (used as a control) was indistinguishable in *lrrkA* KO and WT cells.

A

Gene	DDB Id	Name	Forward primer	Reverse primer	Size (bp)
fol1	G0282461	*grlC*	CATTGTTAACAGCACAAGTTG	CATTAAATCCTTCAACTACCAA	123
fol2	G0272030		GTCACCCTTTCATTCTCATCAG	GCAATTTGCATATATTTTGAATGG	146
fol3	G0293208		GGTTCATGTCAAGGAAGTTGTTC	GTTGATGGATTCCATGATGCAT	146
fol4	G0286015	*gmsA*	GTCACCAACCGAACAACAAA	CATTCCATTTTAATGCTGGAAT	104
fol5	G0271684	*grlA*	GTGGTGATTGGAGTGATATGGG	GAATCTTCAATTGCTTGTTTGG	145
fol6	G0275559		GCAATTACTCGTAATCCAGCAG	CCGAATTTTTCCATTGTCTGTT	137
fol7	G0289485	*vacA*	GAAGCCAATACATTATCATCTG	GTCTTTCAGAGGATTCATTGG	109
fol8	G0272244	*grlG*	CCAAGGAAAAGTAAATGTTGAA	GCCTTCATACATTTTGGCAAT	187
fol9	G0282459	*grlH*	CAGCCAATGTAAATGATATGGG	ACATGCGACAGTATGACCTTG	200
fol10	G0285995	*pdsA*	CCCATCAACAAGAAGATTGTGA	ATTCCAATTCTCTGGTGTATAATAA	143

B

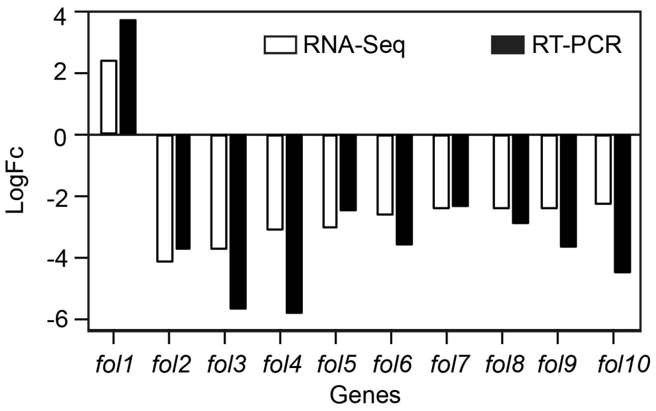

Figure S5. Regulation of gene expression by folate. *D. discoideum* WT cells grown in HL5 were exposed or not to 1mM folate for 4h. RNA-seq analysis allowed the identification of 10 genes for which transcription was significantly altered by exposure to folate. A. For each gene considered (*fol1* to *fol10*), the gene identity and name are indicated, as well as the pair of primers used for RT-PCR and the size of the amplicon. B. For the 10 genes analyzed, RT-PCR analysis confirmed the results obtained by RNA-seq.
